# Comparing Visual Outcomes Between Two Extended Depth-of-Focus Intraocular Lenses in an Asian Population

**DOI:** 10.3390/diagnostics16183024

**Published:** 2026-09-18

**Authors:** Jessica Q. H. Choo, Peng Y. Tan, Younian Yang, Melissa H. Y. Wong

**Affiliations:** Singapore National Eye Centre, 11 Third Hospital Ave, Singapore 168751, Singapore

**Keywords:** extended depth of focus, intraocular lens, PureSee, Vivity, cataract surgery

## Abstract

**Background/Objectives**: To compare the visual outcomes between two extended depth-of-focus intraocular lenses (EDOF IOLs) in an Asian population. **Methods**: This retrospective single-center study included patients with senile cataract undergoing cataract surgery with implantation of either TECNIS PureSee or AcrySof IQ Vivity IOL. Visual and refractive outcomes such as visual acuity (VA), manifest refraction, and mean refractive spherical equivalent were compared up to post-operative month 6. Contrast sensitivity and defocus curves were evaluated monocularly, as well as binocularly if both eyes had the same IOL implanted. **Results:** Forty-nine eyes were evaluated—24 implanted with PureSee and 25 implanted with AcrySof IQ Vivity. The follow-up period was 6.4 ± 1.1 months in the PureSee group and 6.4 ± 1.0 months in the Vivity group (*p* = 1.0). There was no significant difference in postoperative visual outcomes at postoperative month 1 or 6. At post-operative month 6, unaided distance VA was 0.06 ± 0.10 and 0.08 ± 0.14 in the PureSee and Vivity groups, respectively (*p* = 0.519); unaided intermediate VA was 0.21 ± 0.05 and 0.18 ± 0.09, respectively (*p* = 0.192); and unaided near VA was 0.38 ± 0.12 and 0.38 ± 0.12, respectively (*p* = 0.842). Defocus and contrast sensitivity performance were broadly similar, except for Vivity’s defocus being marginally better at 66 cm intermediate distance. At post-operative month 1, 69.2% of Vivity patients (*n* = 13) and 75.0% of PureSee patients (*n* = 4) denied haloes or glare. **Conclusions:** Both EDOF IOLs demonstrated comparable visual performances.

## 1. Introduction

Extended depth of focus (EDOF) intraocular lens implants (IOLs) form a middle ground between monofocal and multifocal IOLs, providing enhanced range of vision through targeting spectacle independence for intermediate vision [1]. This option is often considered in patients that are keen for some form of spectacle independence but are not fit for multifocal IOLs, or if the potential downsides of multifocal IOLs such as glare and haloes cannot be accepted. This is especially important in today’s world, where a lot of time is spent using intermediate vision on digital devices for both leisure and work.

There are many different designs for EDOF IOLs, such as diffractive-based (TECNIS Symfony (Johnson and Johnson Surgical Vision, Irvine, CA, USA [2]) and Zeiss AT LARA (Carl Zeiss Meditec AG, Jena, Germany)), spherical aberration-based (Mini WELL Ready IOL, SIFI S.p.A, Catania, Italy [3] and LuxSmart, Bausch & Lomb GmbH, Berlin, Germany [4]), pinhole effect (IC-8, AcuFocus Inc., Irvine, CA, USA [5]), and wavefront-shaping technology (Vivity IOL, Alcon Laboratories, Fort Worth, TX, USA [6]).

The TECNIS PureSee IOL (Johnson and Johnson Surgical Vision, Irvine, CA, USA) is one of the latest EDOF IOLs with a purely refractive design. The lens is designed to provide an increased vision range including intermediate vision, whilst having distance vision, contrast sensitivity and a dysphotopsia profile similar to a monofocal IOL [7]. In contrast, Vivity IOL functions via a wavefront-shaping technology that is located on the anterior surface of the lens in the central 2.2-mm region of the lens and uses two smooth-surface transition elements that work effectively to alter the travel distance of the light entering the eye, producing a continuous extended range of vision from distance to functional near [6,8]. However, so far there have been no published studies that seek to compare these two EDOF IOLs that employ differing technologies. This study thus seeks to achieve this objective, by comparing these two different IOLs, which adopt varying technologies to achieve the EDOF functionality. Through this study, we found that both EDOF IOLs demonstrated comparable visual performance in our Asian population.

## 2. Materials and Methods

### 2.1. Study Design and Patient Consent Statement

This is a retrospective single-center study conducted in Singapore National Eye Centre. This study was subjected to a SingHealth Institutional Review Board (IRB) waiver. This study adhered to the tenets of the Declaration of Helsinki. Data was anonymized and patient consent was not required from the study projects, as per institutional guidelines and local legislation in Singapore.

### 2.2. Inclusion and Exclusion Criteria

Patients with senile cataract scheduled to have cataract surgery with implantation of either TECNIS PureSee IOL or AcrySof IQ Vivity IOL were included. In total, 49 eyes were included, 24 which were implanted with TECNIS PureSee IOL and 25 with AcrySof IQ Vivity IOL. None of the eyes had other ocular comorbidities besides senile cataracts, including but not limited to prior ocular surgery, corneal irregularity, macular disease, optic nerve disease, or amblyopia.

### 2.3. IOL Description

TECNIS PureSee is a purely refractive, 1-piece soft acrylic aspheric foldable hydrophobic posterior chamber IOL made from SENSAR UV (OptiBlue) material. It has an anterior aspheric surface designed to compensate for average corneal spherical aberration and a posterior refractive surface with a modified refractive design that creates a continuous change in power that modulates the wavefront directing rays to create an extended depth of focus [7]. It is designed for placement in the capsular bag.

AcrySof IQ Vivity is a wavefront-shaping technology “X-WAVE™ Technology” 1-piece soft acrylic aspheric foldable hydrophobic posterior chamber IOL. This technology is deployed on the anterior surface of the lens in the central 2.2-mm region of the lens and uses two smooth-surface transition elements that work effectively to alter the travel distance of the light entering the eye, producing a continuous extended range of vision from distance to functional near [9,10]. It is designed for placement in the capsular bag.

### 2.4. Pre-Operative Evaluation and Surgical Procedure

Pre-operatively, all patients underwent a comprehensive slit-lamp examination with dilated fundal examination to evaluate both the anterior and posterior segment. Corneal tomography was evaluated using Pentacam^®^ (Oculus, Wetzlar, Germany), and optical biometry was performed with IOLMaster 700 (Carl Zeiss Meditec AG, Jena, Germany) and the Barrett Universal II or the Barrett Toric formula was used. The lens factor constant utilized for the PureSee IOL was +2.09, the lens factor constant utilized for the Acrysof Vivity IOL was +1.99, and the intended refractive strategy was to aim for plano. Phacoemulsification was performed by a group of experienced surgeons certified to implant premium IOLs for subjects in this study. Surgeons employed their typical small-incision technique for cataract extraction, siting their main corneal incisions temporally with a 2.65 mm blade if the Stellaris Elite™ (Bausch  +  Lomb, Bridgewater, NJ, USA) was used, or a 2.75 mm blade if the Centurion^®^ Vision System (Alcon Laboratories, Inc.; Fort Worth, TX, USA) was used. IOLs were inserted into the capsular bag with the corresponding validated insertion system. If patients had significant astigmatism, a toric version of the EDOF IOL would be implanted. There was no difference in surgical technique, biometry measurement technique, IOL-calculation formulas, postoperative protocols or patient selection between the two IOL groups.

### 2.5. Outcome Measures

Comparison of monocular uncorrected distance and corrected distance visual acuity (UDVA and CDVA, respectively) and uncorrected intermediate and near visual acuity (UIVA and UNVA, respectively) up to post-operative month (POM) 6 were performed. Distance visual acuity was measured using a logarithmic visual acuity chart (ETDRS; Precision Vision, Woodstock, IL, USA), intermediate visual acuity was measured at 63 cm and near visual acuity was measured at 40 cm. The manifest refraction results and accuracy of mean spherical equivalent refraction to the target were also compared. In addition, contrast sensitivity and defocus curves were evaluated, both monocularly and binocularly if both eyes had the same IOL implanted. Contrast sensitivity was evaluated with the Functional Visual Acuity Test (F.A.C.T.^®^) (Stereo Optical Company Inc., Chicago, IL, USA) under dim lighting. Qualitative evaluation of dysphotopsia was done at POM1. Due to differing time periods of IOL implantation, the Vivity subgroup completed the quality-of-life questionnaire as detailed in Appendix A and the PureSee subgroup completed McAlinden’s Quality of Vision questionnaire as detailed in Appendix B [11].

### 2.6. Statistical Analysis

All statistical analysis was performed using IBM SPSS Statistics for Windows (Version 26) (International Business Machines Corporation, Armonk, NY, USA). A significance level of *p* = 0.05 was applied, and exact two-tailed *p*-values were reported for all statistical tests. T-tests were used for continuous variables and Fisher’s Exact Test for categorical variables. Further analysis was also done to account for within-patient clustering using linear mixed-effects models.

## 3. Results

Forty nine eyes underwent standard phacoemulsification over the period April 2021 to October 2021 for the Vivity group and January to June 2024 for the PureSee group. Twenty four eyes (49.0%) were implanted with PureSee, and twenty five eyes (51.0%) were implanted with AcrySof IQ Vivity IOL. Five patients contributed both eyes in the PureSee group, and four patients contributed both eyes in the Vivity group. In terms of toric versions of IOLs utilized, one toric PureSee IOL was used and 11 toric Vivity IOLs were used. Nevertheless, there was no significant difference in the mean pre-operative corneal cylinder, as detailed in Table 1. The mean ages of patients in the PureSee group and Vivity group were 62.5 ± 8.7 years old and 69.1 ± 7.7, respectively (*p* = 0.033). The percentages of female patients in the PureSee group and Vivity group were 85.7% and 52.9%, respectively (*p* = 0.068). The percentage of Chinese patients in the PureSee group and Vivity group were 64.3% and 82.4%, respectively (*p* = 0.412). The follow-up period was 6.4 ± 1.1 months in the PureSee group and 6.4 ± 1.0 months in the Vivity group (*p* = 1.0). None of the eyes had any pre-existing ocular history other than cataracts. In terms of comparison of pre-operative data, the PureSee group had a significantly more myopic post-operative refractive target compared to the Vivity group (−0.16 ± 0.17 D versus −0.01 ± 0.10 D, *p* = 0.000). There were no other significant differences between the groups. This is further detailed in Table 1.

There was no significant difference in post-operative visual outcomes as described in Table 2. Post-operative month 1 (POM1) UDVA (mean ± SD) (logMAR) was 0.10 ± 0.10 and 0.11 ± 0.11 in the PureSee and Vivity groups, respectively (95% CI −0.066–0.050, *p* = 0.787). POM1 UIVA was 0.15 ± 0.13 and 0.10 ± 0.12, respectively (95% CI −0.030–0.138, *p* = 0.200). POM1 UNVA was 0.38 ± 0.19 and 0.41 ± 0.17, respectively (95% CI −0.146–0.117, *p* = 0.820). POM6 UDVA was 0.06 ± 0.10 and 0.08 ± 0.14, respectively (95% CI −0.114–0.059, *p* = 0.519). POM6 UIVA was 0.21 ± 0.05 and 0.18 ± 0.09, respectively (95% CI −0.018–0.085, *p* = 0.192). POM6 UNVA was 0.38 ± 0.12 and 0.38 ± 0.12, respectively (95% CI −0.074–0.090, *p* = 0.842).

Refractive predictability was high and similar between the two IOLs, as described in Table 2. The MRSE at POM1 was −0.21 ± 0.37 and −0.25 ± 0.41, respectively (95% CI −0.195–0.314, *p* = 0.637). The MRSE at POM6 was −0.14 ± 0.39 and −0.12 ± 0.36, respectively (95% CI −0.189–0.354, *p* = 0.538). There was also good biometry accuracy in both groups—MAE at POM1 was 0.26 ± 0.23 and 0.36 ± 0.30, respectively (95% CI −0.274–0.075, *p* = 0.253). MAE at POM6 was 0.32 ± 0.20 and 0.30 ± 0.24, respectively (95% CI −0.116–0.194, *p* = 0.609). The post-operative spherical equivalent numerical error was 37.5% and 52.0% for the PureSee and Vivity groups, respectively, in the range of −0.25 D to +0.25 D (*p* = 0.310).

**Figure 1:**
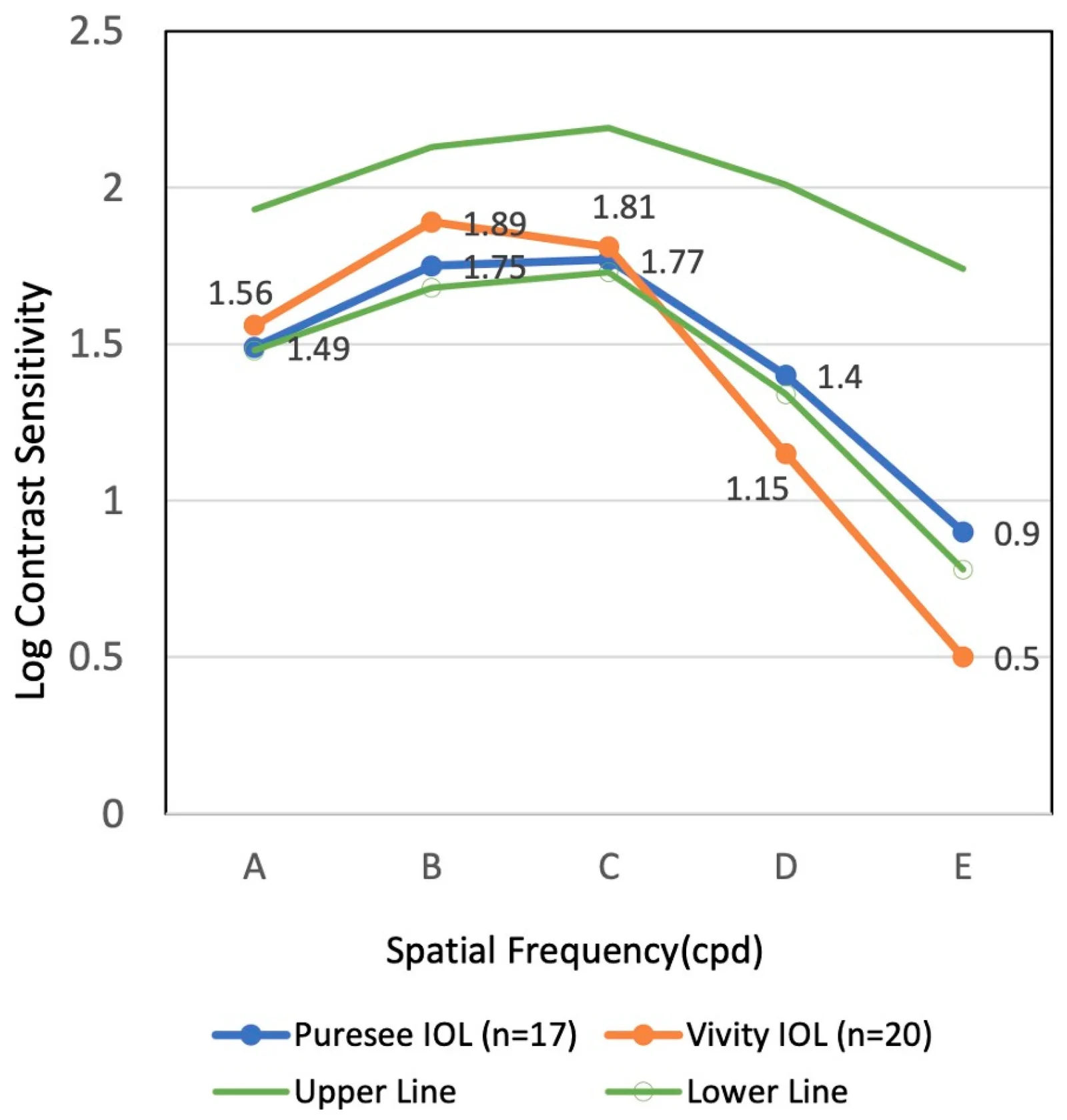
Comparison of contrast sensitivity between operated eye with PureSee vs. Vivity IOL monocularly at post-operative month 6. (A = 1.5 cpd, B = 3 cpd, C = 6 cpd, D = 12 cpd, E = 18 cpd).

**Figure 2:**
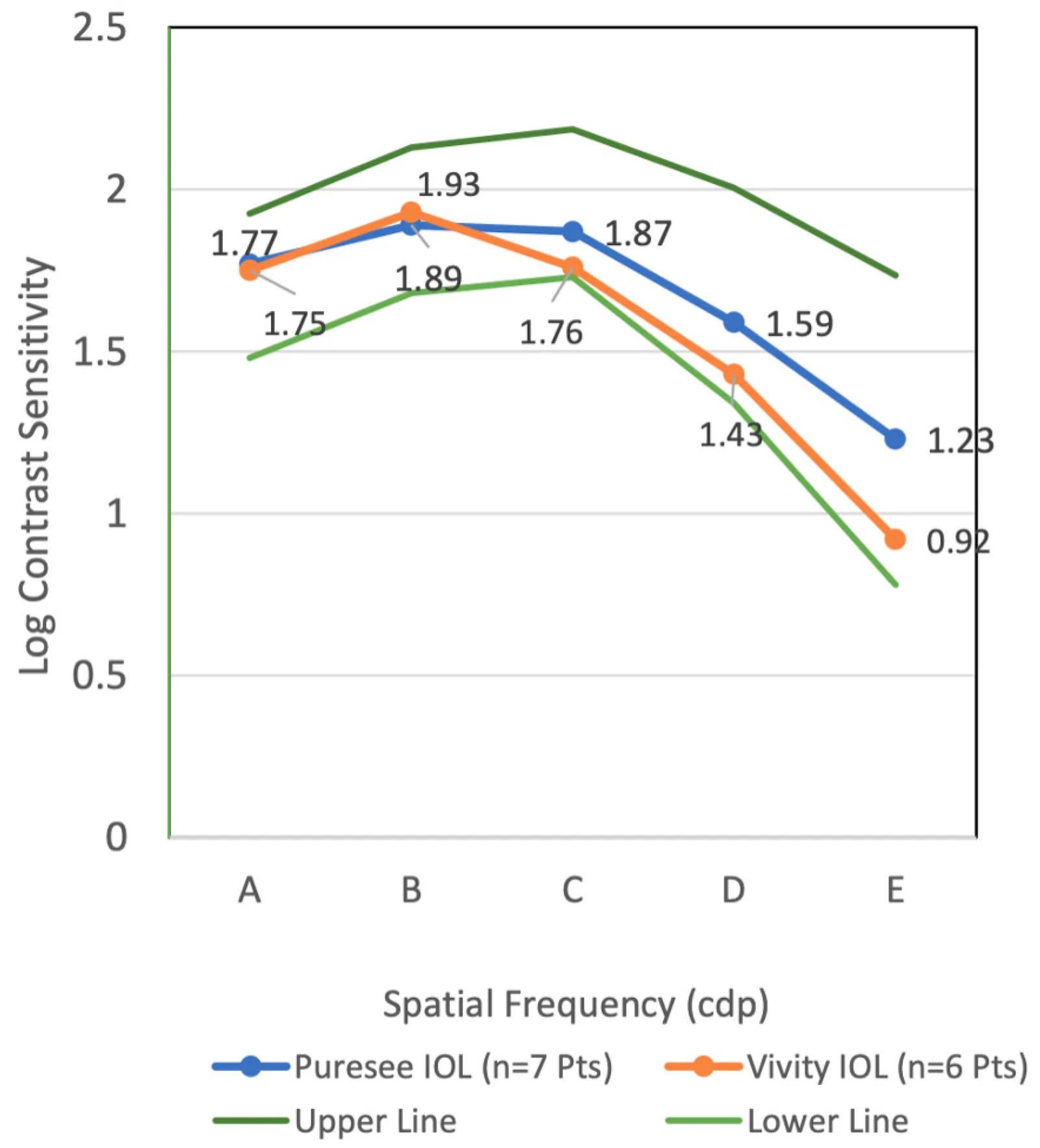
Comparison of contrast sensitivity when eyes were binocularly implanted with either PureSee or Vivity IOL at post-operative month 6. (A = 1.5 cpd, B = 3 cpd, C = 6 cpd, D = 12 cpd, E = 18 cpd).

**Figure 3:**
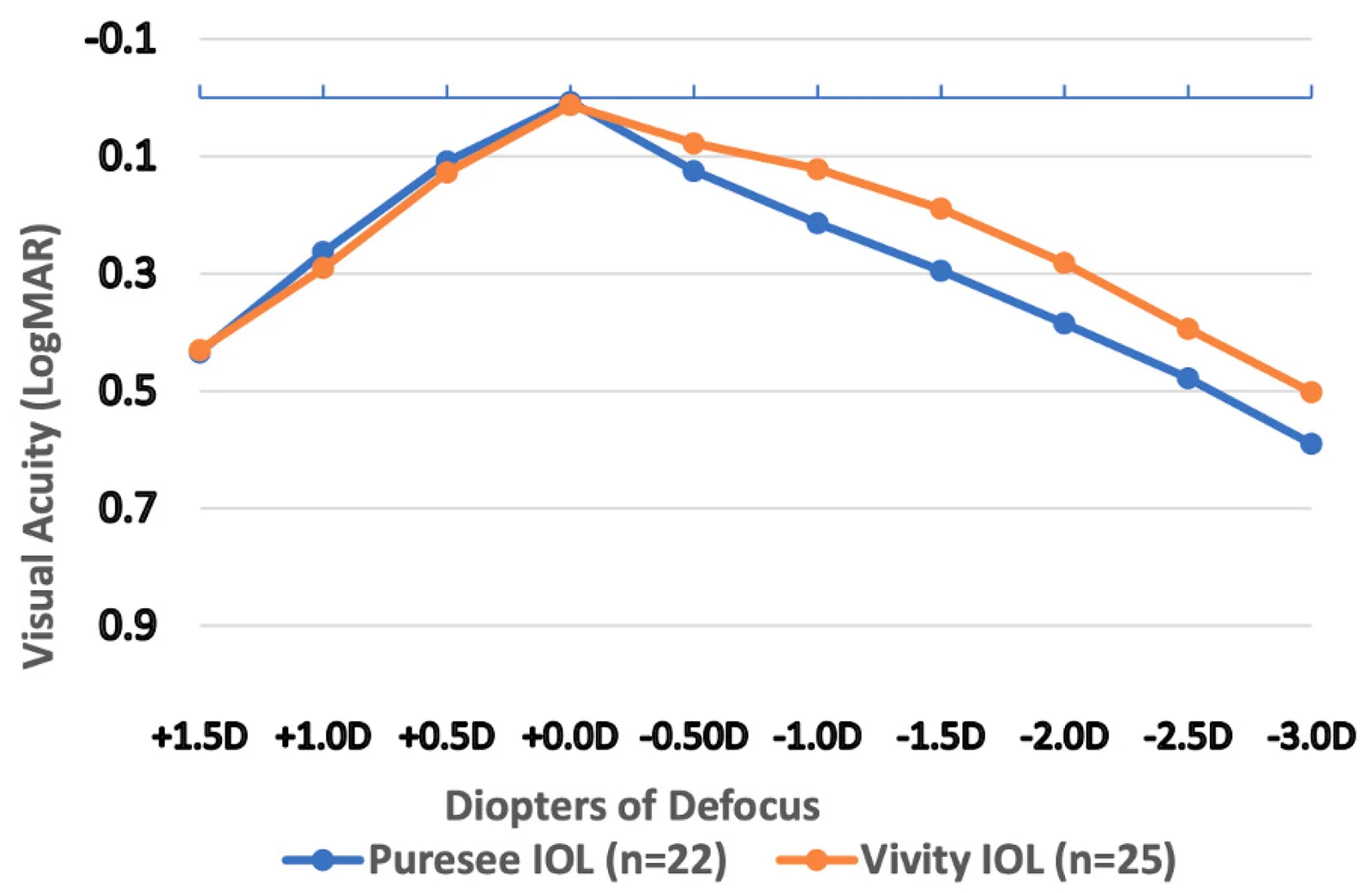
Comparison of defocus between operated eye with PureSee vs. Vivity IOL monocularly at post-operative month 6.

**Figure 4:**
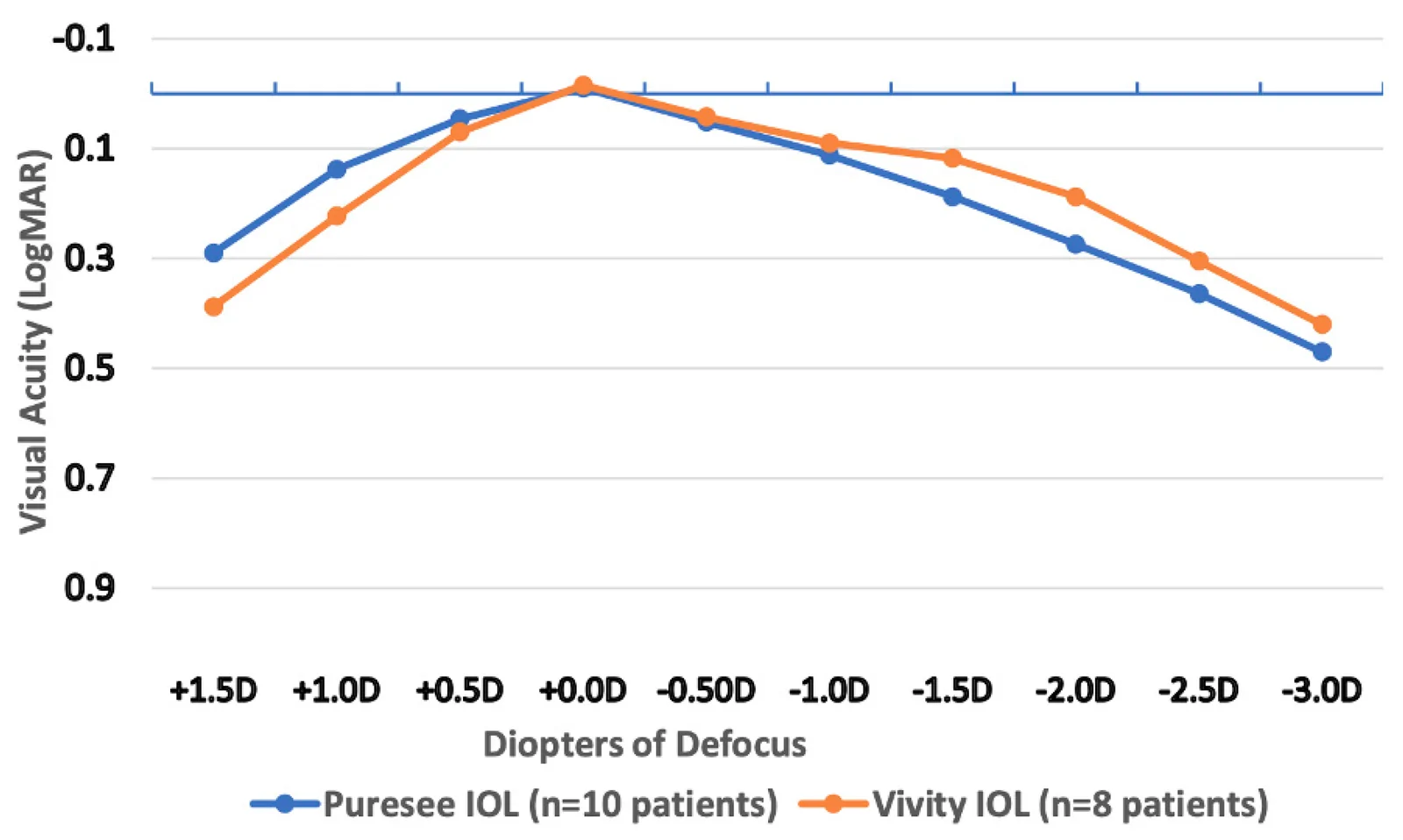
Comparison of defocus when eyes were binocularly implanted with either PureSee or Vivity IOL at post-operative month 6.

As seen in Figure 1, Figure 2, Figure 3 and Figure 4, defocus and contrast sensitivity performance were also measured. Some patients did not comply with the special follow-up visit for defocus and contrast sensitivity measurements; Thus, there was dropout, and the number of eyes recorded was lower than the initial number of eyes enrolled in the study. Nevertheless, it appears that the defocus and contrast sensitivity performance measured were fairly similar between the two groups. At 66 cm intermediate distance, Vivity’s defocus appeared to be marginally better than PureSee, but due to the lack of statistical power, this inference cannot be validated. All patients had no intra-operative complications or post-operative adverse events. In terms of dysphotopsia symptoms, at POM1, 69.2% of Vivity patients (*n* = 13) and 75.0% of PureSee patients (*n* = 4) subjectively denied experiencing any haloes or glare. Not all patients completed the questionnaires due to a failure to attend the special follow-up appointment designated for completion of this questionnaire. Also, the presence or absence of dysphotopsia symptoms was not measured for statistical significance due to the differing use of qualitative questionnaires to enquire about haloes or glare. Thus, these observations in our study population are descriptive in nature and should not be used to suggest comparable dysphotopsia profiles.

## 4. Discussion

Our study also showed that the implantation of either IOL was safe and predictable, with no post-operative adverse events and a low MRSE and MAE in both groups. Moreover, the fact that there was no significant difference in post-operative visual and refractive outcomes between the two IOLs shows that although the EDOF technology can differ, both IOLs are effective at delivering a good visual performance.

There was a significant difference in the planned refractive target spherical equivalent between the two subgroups, with a more myopic target planned in the PureSee group compared to the Vivity group. This is likely due to differing company recommendations on refractive target aims when choosing an IOL power–locally; it is recommended to choose the “first minus” for a PureSee IOL versus the closest to emmetropia or the “first small plus” for a Vivity IOL when aiming for emmetropia from pre-operative biometry. Nevertheless, the MRSE post-operatively ended up statistically similar between the two groups, and the defocus performance was also fairly similar between the two groups. In fact, the Vivity group performed marginally better in terms of defocus performance at 66 cm intermediate distance. A simulation study performed by Niknahad et al. also showed a similar better performance at intermediate focus, albeit with a comparison between Clareon Vivity and PureSee [12]. A future extension could be to conduct an adjusted analysis in view of this significant pre-operative difference with a larger sample size.

As PureSee is a newer EDOF IOL in the market, the published literature on it is much more limited with optical bench or laboratory evaluations. There have not been any clinical studies published at present. A previous study by Song et al. comparing Vivity and Symfony (an older EDOF model from Johnson & Johnson) also showed similar visual and refractive outcomes between both groups [13]. However, in that study, the Symfony group experienced significantly more glare and starbursts than the Vivity group, at 28% and 22%, respectively [14]—this difference was less evident in our study comparing PureSee and Vivity.

For AcrySof IQ Vivity, there are other published studies that have analyzed the visual outcomes of AcrySof IQ Vivity, and the 6-month visual outcomes in the Vivity subgroup showed better outcomes compared to those seen in our study population [6,15]. Nevertheless, this is limited by the fact that these study populations were dissimilar to ours, as they only included non-toric AcrySof IQ Vivity IOL patients who had pre-operative astigmatism of <1.0 D.

One limitation is the relatively modest sample size with slight demographic differences between the two groups. With a small sample size, our study is vulnerable to type II error. Moreover, the patients that received the Vivity IOL were operated on earlier in 2021 compared to the patients that received the PureSee IOL that were operated on in 2024. Thus, there is a risk of selection and temporal confounding. Also, surgeons may inevitably have more experience with such premium IOLs in 2024 compared to in 2021, thus adding further to potential baseline differences between the two subgroups. A further improvement of this study would be to expand our study population with a prospective, controlled study design. With a prospective study protocol, we can do power calculations and utilize a predefined equivalence or non-inferiority framework. Accordingly, with our current study design, failure to identify statistically significant differences does not necessarily establish equivalence or comparability between the lenses. We can also analyze our population over a longer follow-up period to evaluate other factors such as posterior capsular opacification. Moreover, subjective metrics on glares, haloes and QoL evaluation could not be directly compared between the two groups due to differing qualitative questionnaires used to evaluate the subgroups at different time periods, limiting detailed comparison on patient satisfaction and dysphotopsia symptoms and preventing a complete descriptive observation to suggest whether the two IOLs have comparable dysphotopsia profiles or not. Further modifications and enhancements will be explored in future studies.

In conclusion, PureSee and Vivity have demonstrated satisfactory and comparable visual performance in our Singaporean population. Our findings have shown that both IOLs reliably achieved good functional vision with minimal trade-off in optical quality. It would also be of interest in future studies to evaluate how the two IOLs will perform in terms of binocular unaided distance VA, intermediate VA and near VA after binocular implantation, with a further subgroup paired comparison between bilateral aim plano versus mini-monovision.

## Figures and Tables

**Table 1:** Baseline demographics and pre-operative data. CDVA = corrected distance visual acuity. CI = confidence interval.

	TECNIS PureSee™ (*n* = 24)	AcrySof IQ Vivity (*n* = 25)	Difference, PureSee−Vivity	95% CI	*p*-Value
Target SE (mean ± SD) (dioptres)	−0.16 ± 0.17	−0.01 ± 0.10	−0.150	−0.230 to −0.070	<0.001
IOL power (mean ± SD) (dioptres)	18.73 ± 4.77	20.44 ± 2.44	−2.393	−5.392 to 0.606	0.113
Pre-operative axial length (mean ± SD) (millimeters)	24.06 ± 1.34	23.79 ± 0.96	0.540	−0.384 to 1.463	0.241
Pre-operative anterior chamber depth (mean ± SD) (millimeters)	3.13 ± 0.47	2.96 ± 0.50	0.232	−0.141 to 0.605	0.214
Pre-operative monocular CDVA (mean ± SD) (logMAR)	0.32 ± 0.25	0.30 ± 0.40	0.031	−0.182 to 0.244	0.767
Pre-operative corneal cylinder (mean ± SD) (dioptres)	0.69± 0.49	0.83 ± 0.63	−0.084	−0.491 to 0.322	0.675

**Table 2:** Post-operative visual and refractive outcomes. UDVA = uncorrected distance visual acuity. CDVA = corrected distance visual acuity. UIVA = uncorrected intermediate visual acuity. UNVA = uncorrected near visual acuity. MRSE = mean refractive spherical equivalent. CI = confidence interval.

	TECNIS PureSee™ (*n* = 24)	AcrySof IQ Vivity (*n* = 25)	Difference, PureSee−Vivity	95% CI	*p*-Value
Post-operative day 1 UDVA (mean ± SD) (logMAR)	0.24 ± 0.22	0.19 ± 0.16	0.057	−0.065 to 0.180	0.347
Post-operative day 1 CDVA (mean ± SD) (logMAR)	0.17 ± 0.12	0.15 ± 0.13	0.021	−0.055 to 0.097	0.572
Post-operative month 1 UDVA (mean ± SD) (logMAR)	0.10 ± 0.10	0.11 ± 0.11	−0.008	−0.066 to 0.050	0.787
Post-operative month 1 CDVA (mean ± SD) (logMAR)	0.02 ± 0.05	0.04 ± 0.06	−0.019	−0.055 to 0.017	0.295
Post-operative month 1 UIVA (mean ± SD) (logMAR)	0.15 ± 0.13	0.10 ± 0.12	0.054	−0.030 to 0.138	0.200
Post-operative month 1 UNVA (mean ± SD) (logMAR)	0.38 ± 0.19	0.41 ± 0.17	−0.015	−0.146 to 0.117	0.820
Post-operative mean refractive spherical equivalent (MRSE) at month 1 (mean ± SD) (dioptres)	−0.21 ± 0.37	−0.25 ± 0.41	0.059	−0.195 to 0.314	0.637
Mean absolute error (MAE) at month 1 (mean ± SD) (dioptres)	0.26 ± 0.23	0.36 ± 0.30	−0.100	−0.274 to 0.075	0.253
Post-operative month 6 UDVA (mean ± SD) (logMAR)	0.06 ± 0.10	0.08 ± 0.14	−0.028	−0.114 to 0.059	0.519
Post-operative month 6 CDVA (mean ± SD) (logMAR)	−0.02 ± 0.07	0.01 ± 0.07	−0.030	−0.078 to 0.019	0.219
Post-operative month 6 UIVA (mean ± SD) (logMAR)	0.21 ± 0.05	0.18 ± 0.09	0.033	−0.018 to 0.085	0.192
Post-operative month 6 UNVA (mean ± SD) (logMAR)	0.38 ± 0.12	0.38 ± 0.12	0.008	−0.074 to 0.090	0.842
Post-operative mean refractive sphere at month 6 (mean ± SD) (dioptres)	0.15 ± 0.33	0.08 ± 0.41	0.150	−0.118 to 0.417	0.262
Post-operative mean refractive cylinder at month 6 (mean ± SD) (dioptres)	−0.57 ± 0.45	−0.40 ± 0.26	−0.135	−0.382 to 0.113	0.275
Post-operative mean refractive spherical equivalent (MRSE) at month 6 (mean ± SD) (dioptres)	−0.14 ± 0.39	−0.12 ± 0.36	0.083	−0.189 to 0.354	0.538
Mean absolute error (MAE) at month 6 (mean ± SD) (dioptres)	0.32 ± 0.20	0.30 ± 0.24	0.039	−0.116 to 0.194	0.609

## Data Availability

The original contributions presented in this study are included in the article. Further inquiries can be directed to the corresponding author.

## Abbreviations

The following abbreviations are used in this manuscript:

CDVA	corrected distance visual acuity
CI	confidence interval
EDOF	extended depth of focus
IOL	intraocular lens
IRB	Institutional Review Board
MAE	mean absolute error
MRSE	manifest refraction spherical equivalent
POM1	postoperative month 1
POM6	postoperative month 6
UDVA	uncorrected distance visual acuity
UIVA	uncorrected intermediate visual acuity
UNVA	uncorrected near visual acuity

## Appendix A

### Quality-of-Life Questionnaire for Vivity Subgroup

**Table A1:** Part 1 of quality-of-life questionnaire for Vivity subgroup.

Question	All the Time	Most of the Time	Some of the Time	A Little of the Time	None of the Time
Do you experience glares and halos?					
Is your driving affected at night?					
Do you have to adjust your reading distance now?					
Are you happy/satisfied with your visual results?					
Do you have to modify any aspect of your life after the operation? If so, please specify. ___________					

**Table A2:** Part 2 of quality-of-life questionnaire for Vivity subgroup.

Activity (NA If You Do Not Engage in the Activity)	Percentage of Time Using Spectacles (0–100%)	Modifications
Threading a needle		
Writing at the desk		
Shaving/putting on makeup		
Reading a book/newspaper		
Using the iPad/phone/any tablet		
At a desktop computer		
Playing golf		
Driving in the day		
Driving in the evening/night		
Looking at the menu in a restaurant		
Grocery shopping		
Looking for your seat in a cinema		

## Appendix B

### McAlinden’s Quality of Vision Questionnaire for PureSee Subgroup

Quality of Vision Questionnaire

1. How often do you experience glare?
2. How severe is the glare?
3. How bothersome is the glare?
4. How often do you experience haloes?
5. How severe are the haloes?
6. How bothersome are the haloes?
7. How often do you experience starbursts?
8. How severe are the starbursts?
9. How bothersome are the starbursts?
10. How often do you experience hazy vision?
11. How severe is the hazy vision?
12. How bothersome is the hazy vision?
13. How often do you experience blurred vision?
14. How severe is the blurred vision?
15. How bothersome is the blurred vision?
16. How often do you experience distortion?
17. How severe is the distortion?
18. How bothersome is the distortion?
19. How often do you experience double or multiple images?
20. How severe are the double or multiple images?
21. How bothersome are the double or multiple images?
22. How often do you experience a fluctuation in your vision?
23. How severe is the fluctuation in your vision?
24. How bothersome is the fluctuation in your vision?
25. How often do you experience focusing difficulties?
26. How severe are the focusing difficulties?
27. How bothersome are the focusing difficulties?
28. How often do you experience difficulty judging distance or depth perception?
29. How severe is the difficulty judging distance or depth perception?
30. How bothersome is the difficulty judging distance or depth perception?
